# Effects of a digitally enabled cardiac rehabilitation intervention on risk factors, recurrent hospitalization and mortality

**DOI:** 10.1093/ehjdh/ztaf043

**Published:** 2025-04-29

**Authors:** Justin Braver, Thomas H Marwick, Agus Salim, Dulari Hakamuwalekamlage, Catherine Keating, Stephanie R Yiallourou, Brian Oldenburg, Melinda J Carrington

**Affiliations:** Baker Department of Cardiometabolic Health, Faculty of Medicine, Dentistry and Health Sciences, The University of Melbourne, Parkville, Victoria, Australia; Community Prevention and Cardiac Research, Baker Heart and Diabetes Institute, 75 Commercial Road, Melbourne, VIC 3004, Australia; Clinical Hub, Medibank, Melbourne, Australia; Baker Department of Cardiometabolic Health, Faculty of Medicine, Dentistry and Health Sciences, The University of Melbourne, Parkville, Victoria, Australia; Community Prevention and Cardiac Research, Baker Heart and Diabetes Institute, 75 Commercial Road, Melbourne, VIC 3004, Australia; Community Prevention and Cardiac Research, Baker Heart and Diabetes Institute, 75 Commercial Road, Melbourne, VIC 3004, Australia; Community Prevention and Cardiac Research, Baker Heart and Diabetes Institute, 75 Commercial Road, Melbourne, VIC 3004, Australia; Clinical Hub, Medibank, Melbourne, Australia; Centre for Health, Exercise and Sports Medicine, Department of Physiotherapy, The University of Melbourne, Parkville, Victoria, Australia; Turner Institute for Brain and Mental Health, School of Psychological Sciences, Monash University, Clayton, Victoria, Australia; Community Prevention and Cardiac Research, Baker Heart and Diabetes Institute, 75 Commercial Road, Melbourne, VIC 3004, Australia; School of Psychology and Public Health, La Trobe University, Melbourne, Australia; Baker Department of Cardiometabolic Health, Faculty of Medicine, Dentistry and Health Sciences, The University of Melbourne, Parkville, Victoria, Australia; Community Prevention and Cardiac Research, Baker Heart and Diabetes Institute, 75 Commercial Road, Melbourne, VIC 3004, Australia

**Keywords:** Digital health, Telerehabilitation, Cardiac rehabilitation, Coronary artery disease, Cardiovascular disease

## Abstract

**Aims:**

Cardiac rehabilitation (CR) programmes are effective, but they are underutilized. Digitally enabled CR programmes (DeCR) offer alternative means of healthcare delivery. We aimed to assess the effects of a DeCR programme on cardiovascular risk factors and healthcare utilization.

**Methods and results:**

In this observational cohort study that used propensity score matching, privately insured Australian patients, recruited nationally following a cardiac hospitalization, were given a digital app and received weekly telehealth consultations. Risk factors were assessed before and after the intervention. Propensity scoring methods were used to compare differences in 30-day, 90-day, and 12-month rehospitalizations, hospital-days, and mortality rates in the DeCR group with patients who undertook: (i) usual care (*n* = 266) or (ii) face-to-face CR (F2F-CR, *n* = 115). Overall, 172 intervention patients (70% men, age 68 ± 10 years, 36% living in regional/remote areas) were enrolled (59% agreed to participate and 91% completed final follow-up). The DeCR group had significant improvements in most risk factors. Rehospitalization and mortality rates were similar between the DeCR group and both comparison groups at all time points (all *P* > 0.05). Patients in the DeCR group spent significantly fewer days in hospital compared with usual care within 30-days (*P* = 0.026), 90-days (*P* = 0.003), and 12-months (*P* = 0.04) post-discharge. Cardiac-related rehospitalization bed days were reduced at 30-days (*P* = 0.005) and 90-days (*P* = 0.017) but not 12-months (*P* = 0.20). There were no group differences between DeCR and F2F-CR across any outcomes (all *P* > 0.05).

**Conclusion:**

DeCR was associated with lower healthcare utilization than usual care, yet comparable compared with F2F-CR. DeCR represents a suitable option for cardiac patients post-discharge.

**Lay Summary:**

We investigated whether a digitally enabled cardiac rehabilitation (DeCR) programme, delivered to patients following a heart disease hospitalization, improved patients’ cardiovascular disease risk factors and whether they had a reduction in rehospitalizations, spent fewer days in hospital and improved survival compared with matched controls who undertook either face-to-face cardiac rehabilitation (F2F-CR) or usual care.

• DeCR was associated with similar healthcare utilization outcomes compared with F2F-CR. This suggests that the potential benefits of DeCR may be comparable. Additionally, DeCR programmes create an opportunity for patients to choose the style of CR to undertake and have an advantage of broader access.

• The DeCR group spent significantly fewer readmission days in hospital compared with the usual care group, which may reflect differences in the nature of rehospitalizations when they occur.

## Graphical Abstract

**Graphical Abstract:**
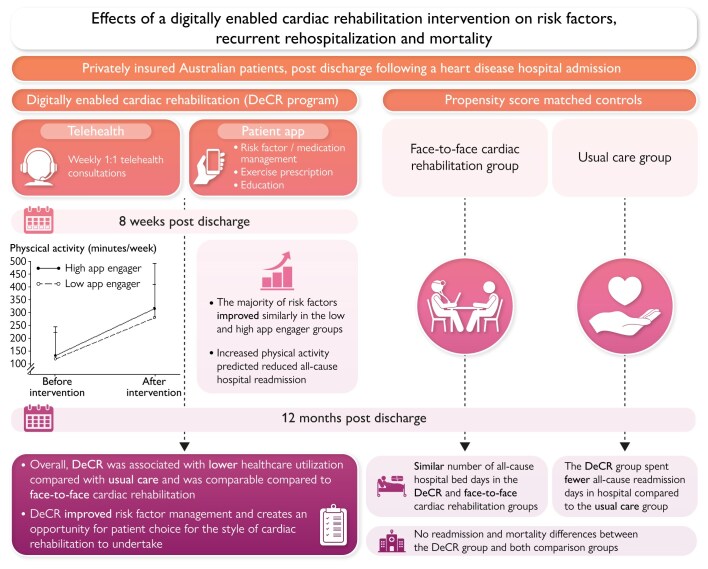

Key Learning Points

1. What is already known
  - Cardiac rehabilitation (CR) programmes are effective in improving risk factor profiles, quality of life and reducing rehospitalization and deaths.
  - Despite the tangible benefits, many barriers impede participation in face-to-face programmes resulting in most eligible patients not undertaking CR.
  - Digitally enabled CR programmes (whereby care is delivered virtually, using digital technologies such as the internet, wearable devices and mobile applications) offer an alternative approach to healthcare delivery and provide expanded options and viable alternatives for patients who face obstacles to participating in traditional face-to-face programmes, particularly those residing in regional or remote areas. However, despite their potential to enhance access, they may introduce other obstacles that require additional support to overcome, such as language barriers when not translated, the need for access to digital technology and a requisite level of digital literacy. Albeit, evidence regarding their potential benefits, including their association with healthcare utilization remains limited.
2. What this study adds
  - The current study sought to determine whether a digitally enabled CR programme, delivered nationally in a real-world setting, improves cardiovascular risk factors and reduces rehospitalization, hospital bed-days, and mortality compared with traditional face-to-face CR and usual care.
  - Results showed that following the completion of the intervention, there were improvements across most cardiovascular risk factors, of which increased physical activity predicted reduced readmission risk at 12 months. Furthermore, patients who completed the digitally enabled CR programme had similar healthcare utilization outcomes compared with patients who undertook face-to-face CR, but favourable outcomes compared with the usual care group. These associations do not infer causation however and there is a need for further investigation via randomized controlled trials.
  - This was a national and entirely remotely delivered CR programme in Australia. Findings provide support for integrating new technologies into CR practice.

## Introduction

Following a cardiac event or procedure, current guidelines recommend that patients attend a secondary prevention cardiac rehabilitation (CR) programme,^1^ which typically entails 11 sessions over a 7 to 8 week period in Australia.^2^ The benefits of CR programmes have been apparent for more than half a century^3^ in improving risk factor profiles, quality of life and reducing rehospitalization and deaths.^4,5^ Despite the demonstrable benefits, many barriers impede participation in face-to-face programmes, resulting in >50% of eligible patients not undertaking CR worldwide,^6^ and between 60% and 90% of patients not participating in a CR programme in Australia,^7^ Europe,^5,8^ and America.^9^ Failure to participate in CR contributes to persisting high rates of unplanned rehospitalizations^10^ with consequent financial burdens, as hospitalization expenditure represents a major driver of cost.^11^

To remedy poor uptake and low participation in traditionally delivered CR programmes, digitally enabled CR programmes (DeCR—whereby care is delivered virtually, using digital technologies such as the internet, wearable devices, and mobile applications^12^) offer an alternative approach to delivery and are endorsed for use in clinical practice.^1^ Digitally enabled health services provide a new opportunity by removing barriers to access and allowing clinicians to remotely monitor, track, and communicate (in real-time and/or asynchronously) with their patients.^12,13^ Prior evidence investigating the virtual delivery of CR, via a mobile application called SmartCR, showed higher uptake and completion rates and improved risk factor management compared with traditional CR.^14^ However, the style of DeCR services have largely been implemented without robust scientific evidence about their effects in real-world settings.^12,13,15^ Moreover, there is limited evidence regarding their impact on healthcare utilization and survival.^1,16^

Therefore, we aimed to evaluate the benefit of a DeCR programme delivered virtually via telehealth and supplemented by the SmartCR mobile application (called, ‘Heart Health at Home’). We extended upon prior research using the same mobile app^14^ by investigating associations between our DeCR programme and longer-term hospital utilization and mortality compared with both face-to-face CR (F2F-CR) or usual care, following a heart disease hospitalization. We hypothesized that our intervention would be associated with healthy lifestyle changes and improvements in clinical risk factors. We also anticipated that ‘Heart Health at Home’ would be associated with reduced hospital readmissions, hospital bed days, and mortality compared with usual care and traditional F2F-CR programmes.

## Methods

### Study design

This was an observational cohort study that implemented propensity score matching to estimate the potential benefit of the ‘Heart Health at Home’ DeCR programme in Australia. Firstly, a prospective cohort study was conducted from October 2019 to February 2021, and thereafter a propensity matched cohort study was performed via analysis of private hospital claims data. Using propensity score matching methods, two concurrent control groups were established to compare the ‘Heart Health at Home’ group with patients who undertook, following a cardiac hospitalization, either: (i) F2F-CR (typically participation in a group setting within a hospital or community centre) or (ii) usual care, (a natural sample of patients who were eligible for CR but unlikely participated in CR as they did not have a CR insurance claim). Once the statistically matched control groups were created, follow-up hospital admission claims data were collected for 12 months to March 2022 for all three groups. The groups were then compared to determine differences in recurrent hospitalizations, mortality and days spent in hospital, within 12 months post their index admission.

We used a propensity score matched cohort design because we sought to extend on a previous randomized controlled trial^14^ by implementing the programme nationally, in an uncontrolled real-world setting, and assessing its impact on hospital utilization and mortality.

The study was approved by the Alfred Hospital Ethics Committee (Project Number: 321/21) and the trial is registered with ClinicalTrials.gov (Identifier NCT06813482). All participants provided consent to participate in ‘Heart Health at Home’ and for their de-identified information to be shared with an independent organization for evaluation. A waiver of consent to access the de-identified personal and health information for matched controls was also approved by the Alfred Hospital Ethics Committee.

### Participants

#### ‘Heart Health at Home’ intervention cohort

Patients aged over 18 years who held private health insurance with one of Australia's largest private health insurers (Medibank; 3.7 million members) at a level that included cover for hospital treatment and CR were eligible. Patients were suitable for inclusion if they were discharged from hospital between October 2019 and November 2020 with a cardiovascular diagnosis and/or procedure eligible for CR, as defined by the National Heart Foundation of Australia (see Supplementary material online, *Table S1*).^17^ Patients were from any State or Territory in Australia and had to be able to give written consent to participate. We excluded patients who: (i) had heart failure (due to the potential for more specialized care which SmartCR was not designed to provide); (ii) were attending an alternate CR programmes for the corresponding index event and; (iii) did not have access to a smart phone and internet connection.

### Selection procedures

The insurer contacted potentially eligible patients via telephone call for recruitment into the ‘Heart Health at Home’ programme. Patients could also self-nominate to participate by contacting the insurer or were referred by a physician within 30 days after being discharged from their index admission. To identify eligible patients, we used national private hospital claims data that documented patient encounters for all procedures performed as per the Australian Classification of Health Intervention and recorded primary diagnoses according to the International Classification of Diseases 10th revision-Australian Modification from all private hospitals across Australia. Codes used to define eligible cardiovascular diagnoses and procedures are provided in Supplementary material online, *Table S2*.

#### Propensity score matched controls

Propensity score matched controls were selected from hospital admission claims data. Each intervention patient was matched with one patient who completed F2F-CR and two patients in the usual care group (see also Statistical Analysis section).

### Study procedures

**Figure 1:**
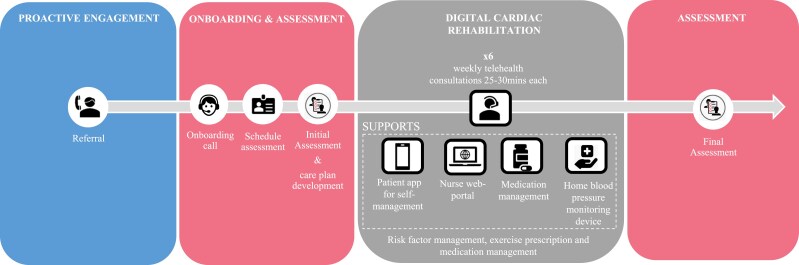
‘Heart Health at Home’ model of care.

‘Heart Health at Home’ (*Figure 1*) was an 8-week remotely delivered CR programme entailing an initial assessment (week 1) followed by 6 weeks participation in a DeCR programme (weeks 2–7) and then a final assessment (week 8). The programme was delivered by Amplar Health, an Australian health services organization that is a subsidiary company of Medibank. The emphasis was to provide secondary prevention of cardiovascular disease (CVD) care^17^ by supporting behaviour change for a healthier lifestyle and diet (including increased physical activity), optimizing medication adherence and enhancing mental health and quality of life, via the following modalities:

1. Telehealth—cardiac nurse telephone coaching to provide patient education and support to enhance risk factor management.
2. Mobile application and nurse web portal—SmartCR (Cardihab®, Brisbane, Australia) incorporates the core components of phase 2 CR with a clinician/nurse web portal to view and manage patient data and provide consultations throughout the intervention. SmartCR captures self-reported health (e.g. blood pressure, stress) and physical activity metrics and provides prompted educational materials (e.g. on symptoms and disease management) via links to audio/video files, written articles and medication management. The SmartCR physical activity programme automatically prompted individuals to gradually increase their walking distance from 5 min twice a day in the first week, reaching a maximum of 30 min by week 6, without prescription of an individualized programme or incorporating any other exercise types (such as resistance/strength training, stretching, and balance exercises). Within an Australian context, clinical guidelines for CR clinicians acknowledge that physical activity is possible to be assessed, prescribed and performed without in-person delivery, so long as it is light to moderate intensity. In contrast to Europe and America, a medical review for light intensity physical activity is not essential. Prior to using the app, nurses undertook a detailed clinical history and used their clinical judgement (to identify higher risk patients) for the suitability of the physical activity programme. If in doubt, it was recommended that participants seek medical advice before commencing the physical activity programme. Nurses provided support and empowered participants to implement their symptom management action plan if required during unsupervised physical activity sessions.

At the start of the programme, participants received an initial assessment via telephone by a nurse to collect self-reported baseline information, develop personalized care plans and to provide guidance for installing the app. Nurses aimed to build patient rapport by actively listening to their concerns, feelings and needs, demonstrating empathy and understanding and involving them in care decisions, as the basis to implementing the intervention. Subsequently, six weekly one-on-one telehealth sessions of 25 to 30 min duration were provided by the same cardiac nurse to reinforce the education topics available via the SmartCR app, encourage motivation and to provide support and positive reinforcement for healthy behaviour changes. At the completion of the DeCR programme, a final telehealth consultation was conducted to collect follow-up information.

### Study data

#### ‘Heart Health at Home’ intervention cohort

Before and after (excluding demographic information) completing the DeCR programme, self-reported measures were assessed by telephone and used for analysis including:

1. Demographic information—age, sex, ethnicity, employment, living situation, location, and geographic based socioeconomic status^18^ (higher scores are less disadvantaged relative to lower scores).
2. Risk factors—blood pressure using an Omron HEM-7121 (Omron Healthcare Co. Ltd, Kyoto, Japan) automated device given to patients to record one daily measurement at around the same time in the day after sitting quietly for 5 min, height and weight for calculation of body mass index, cigarette smoking, diet via a 9-item questionnaire developed by a Medibank and Baker Institute working group based on dietary guidelines (higher scores [range 9,28] denote better diet quality), alcohol (total standard drinks per week) and physical activity (total minutes per week).
3. Health and lifestyle behaviours—medication adherence via the 4-item Morisky Medication Adherence Scale^19^; functional capacity via the 12-item Duke activity status index^20^; psychological distress via the Kessler psychological distress scale (K10)^21^; health status via the EQ-5D-5L (index value-Australia)^22^ and patient engagement in managing their health via the 13-item patient activation measure (PAM).^23^ A higher K10 score represents greater distress whereas higher scores for all other instruments represent better health states.

The SmartCR app tasks that were assessed as completed or not completed for all patients included daily blood pressure and stress levels, twice daily walking data, weekly weight measurements, and personalized education topics. For select individuals with diabetes or high risk alcohol consumption, blood glucose concentration and alcohol intake tasks were only assessed if assigned to patients as a goal by the nurses at the initial assessment when developing personalized care plans. Values of the health (e.g. blood pressure) and physical activity metrics inputted into the app were not used for analysis, rather the action of recording the task as being completed was used in analyses to measure app engagement. Patient engagement for tasks set within the SmartCR app was determined by the number of tasks marked as completed, divided by the number of tasks assigned during the intervention period. Medication engagement via the app was calculated by the number of medication reminders marked as taken or not taken, divided by the total number of medication reminders sent over the intervention period. A composite variable to assess overall app engagement was created by combining responses to tasks assigned and medication reminders. This measure was used to classify patients as low app engagers (<40%) or high app engagers (≥40%) based on the overall median (39%) and mean (40%) app engagement score. This approach aligns with other studies that evaluated patient engagement with mobile apps for the management of CVD.^24^

#### Hospital admission data (‘Heart Health at Home’ intervention and control groups)

Index hospital admission data were collected for all patient groups and included date of admission and discharge, index procedure information, sociodemographic data, CVD condition, smoking history, past history of diabetes, chronic kidney disease, and previous acute myocardial infarction. The applicable health determinants were used to calculate the Charlson comorbidity index^25^ and risk of readmission via the PEGASUS-TIMI score, as defined by age >65 years, eGFR <60 mL/min/1.73 m^2^, prior acute myocardial infarction, multi-vessel coronary artery disease, and diabetes.^26^

#### Propensity score matched controls

We used propensity score matching to create statistically matched control groups. For matched pairs only, we then compared rate of readmission and their duration as well as mortality rates of the ‘Heart Health at Home’ group with the outcomes of individuals who completed either F2F-CR or usual care. Post-discharge outcomes assessed were 30-day, 90-day, and 12-month all cause and cardiac-related readmissions, total hospital bed days, and 30-day and 12-month all-cause mortality.

### Statistical analysis

Continuous variables are reported as mean ± standard deviation or median with inter-quartile range, as appropriate. Discrete variables were assessed by frequencies and percentages. To evaluate the usefulness of ‘Heart Health at Home’ and the influence of app engagement on the change in risk factors, including health and lifestyle behaviours, the average at each time point (before and after the intervention) was compared according to level of app engagement (low vs. high app engagers) in a two-way ANOVA. The χ^2^ tests were applied for categorical variables. Associations with hospital readmission in the ‘Heart Health at Home’ CR group were assessed by logistic regression analysis. Candidate variables included index hospitalization information, clinical risk, demographics, app engagement metrics, baseline, and change in health outcome measures.

#### Propensity score matched controls

For analyses involving propensity score matching, the following baseline characteristics were used to estimate the propensity score using logistic regression: index admission CVD procedure or diagnosis, Charlson comorbidity index, PEGASUS-TIMI readmission risk score,^26^ gender, year of hospital admission and gender × year of admission interaction. The choice of propensity matched variables was based on the availability of private health insurance hospital admission data and selected by the study cardiologist (T.H.M.) in consideration of important confounders that influence the decision to receive CR and variables that are related to the health outcome measures. Age was not included as a separate covariate in the propensity score model since it was already accounted for within the PEGASUS-TIMI criteria and was not associated with treatment allocation when the PEGASUS-TIMI score is already in the model. Discounting age also enhanced the number of matches in the propensity score matching process. Similarly, socioeconomic status, as defined by the IRSD, was not included in the propensity score model because it was not statistically associated with treatment allocation after accounting for the other variables already in the model. We matched each ‘Heart Health at Home’ participant with one individual who completed F2F-CR and two individuals in the usual care group. To be considered as a match, participants were required to have the same primary reason for the index admission and their propensity score needed to fall within ±0.01 (for those who did F2F-CR) and ±0.05 (for those in usual care) of the index ‘Heart Health at Home’ participant. These thresholds were chosen to balance precision for like matches with maintaining a sufficient number of matched pairs. Individuals that were matched were considered as one stratum. A mixed-effect zero-truncated negative binomial model was used to estimate the average bed day ratio between the groups. This was used to address the variability in bed days, including extended bed days. Mixed-effect logistic regression was performed to estimate the mortality differences between the groups and mixed-effect Poisson regression was calculated to determine group effects on the number of readmissions, which also accounted for patients with multiple readmissions. The mixed-effect models were fitted using the R packages lme4^27^ and glmmTMB.^28^ All other statistical analyses were performed using IBM SPSS Statistics 26 (SPSS Inc., Chicago, Illinois, USA). All statistical hypothesis testings were two-sided at the nominal level α = 0.05.

## Results

**Figure 2:**
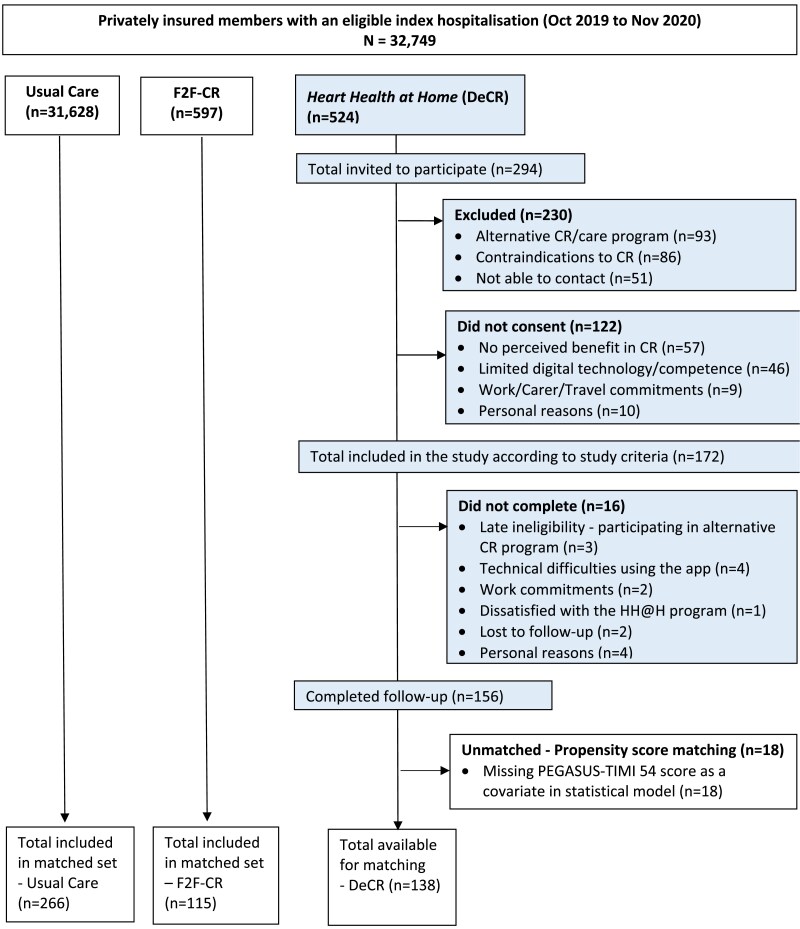
‘Heart Health at Home’ study STROBE flow chart for intervention cohort and propensity score matched controls.

*Figure 2* shows the study STROBE flow chart for the intervention cohort and propensity score matched controls. For the cohort study, of 524 patients assessed for eligibility, 44% (*n* = 230) did not meet the key requirements for the study primarily due to alternative CR preferences, contraindications to CR or they were unable to be contacted. Of 294 patients who had the chance to join the study and were screened to participate, 172 patients (59%) consented to participate of whom 156 (91%) completed the programme (mean intervention exposure 7 weeks) and had follow-up data for final analysis. For propensity score matching, there were an additional 18 participants who completed the ‘Heart Health at Home’ programme that were unmatched due to a missing PEGASUS-TIMI score as a covariate in the statistical model. Overall, there were 138 patients available who completed the ‘Heart Health at Home’ programme to match with 266 from 31 628 patients who completed usual care, and 115 from 597 patients who completed F2F-CR.

### Patient characteristics at baseline

Baseline hospital admission, demographic and clinical characteristics are shown in *Table 1*. Patients were predominantly male (70%) with a mean age of 68 years (63% aged ≥65 years). The majority of patients resided in Victoria (36%), Queensland (28%) or NSW/ACT (24%), and few lived alone (17%). Approximately one third of patients remained employed (full-time or part-time basis) were from regional or rural locations, and located in lower socioeconomic areas (bottom 50th percentile) (each 36%).

**Table 1:** Patient characteristics in the intervention cohort at baseline

	Heart Health at Home (DeCR) (*n* = 172)
Age, ≥65 years (*n*, %)	109 (63)
Male (*n*, %)	120 (70)
Living region (*n*, %):	
ACT or NSW	42 (24)
VIC	61 (36)
QLD	48 (28)
SA	6 (3)
WA	10 (6)
TAS	5 (3)
NT	0 (0)
Socio-economic conditions (*n*, %): IRSD, lowest 50th percentile	61 (36)
Employed, full-time or part-time^a^	61 (36)
Lives alone	29 (17)
Living location, regional or rural	61 (36)
Index admission (*n*, %):	
Acute coronary syndrome:	103 (60)
STEMI	6 (6)
NSTEMI	15 (15)
Unstable angina	82 (80)
Atrial fibrillation	41 (24)
Permanent pacemaker/AICD	21 (12)
Valvular heart disease	7 (4)
Revascularization procedure:	
Angioplasty	48 (47)
Coronary artery bypass graft	27 (26)
No revascularization/medical management	27 (26)
Unclassified	1 (1)
Length of stay, days (median, IQR)	2 [1,6]
Clinical risk (*n*, %)^a^	
Smoking, last 5 years (*n* = 169)	58 (34)
Multi-vessel coronary artery disease (*n* = 138)	32 (23)
Diabetes (*n* = 155)	29 (19)
Irregular heart rhythm	65 (48)
Chronic kidney disease, stage 3–5 (*n* = 138)	2 (1)
Charlson comorbidity index (median, IQR)	1 [0,2]
Charlson comorbidity index >1—*n* = 168	85 (51)
Re-admission risk, PEGASUS-TIMI 54	
High (≥5)—*n* = 138	27 (16)

DeCR, digitally enabled cardiac rehabilitation; IRSD, index of relative socio-economic disadvantage; STEMI, ST segment elevation myocardial infarction; NSTEMI, non-ST segment elevation myocardial infarction; AICD, automated implantable cardioverter defibrillator.

^a^Missing cases: employed (*n* = 2); smoking (*n* = 3); multi-vessel coronary artery disease (*n* = 34); diabetes (*n* = 17); irregular heart rhythm (*n* = 36); chronic kidney disease (*n* = 34); Charlson comorbidity index (*n* = 4); PEGASUS-TIMI 54 (*n* = 34).

The key reason for the index admission was for an acute coronary syndrome (60%) and 24% presented with atrial fibrillation. Of the acute coronary syndrome type, 20% presented with a ST-elevation myocardial infarction or non-ST-segment elevated myocardial infarction and the majority were managed with a revascularization procedure by either angioplasty (47%) or coronary artery bypass grafting (26%). The median length of stay for the index hospitalization was two days [IQR 1,6 days] and 16% of patients were deemed high risk for hospital readmission as per PEGASUS-TIMI 54 criteria.

One-third of participants smoked cigarettes in the last 5 years (2 current smokers), half had an irregular heart rhythm, one-quarter had multi-vessel coronary artery disease and approximately one in five patients had diabetes.

### App engagement

The overall mean app engagement was 40%; this comprised 47% for achievement of assigned tasks and 37% for responding to medication reminders. For medication management exclusively, of 121 (70%) patients who entered their medications into the app, the average number entered was 6 ± 3 medications entailing 7 ± 4 daily reminders. The level of app engagement varied according to the type of task; weekly weight measurement tasks were most successfully achieved by patients (57%), followed by blood glucose tasks (54%), education tasks (50%), alcohol and blood pressure tasks (each 48%), and finally walking and stress level tasks (each 45%).

### ‘Heart Health at Home’ programme effects

**Figure 3:**
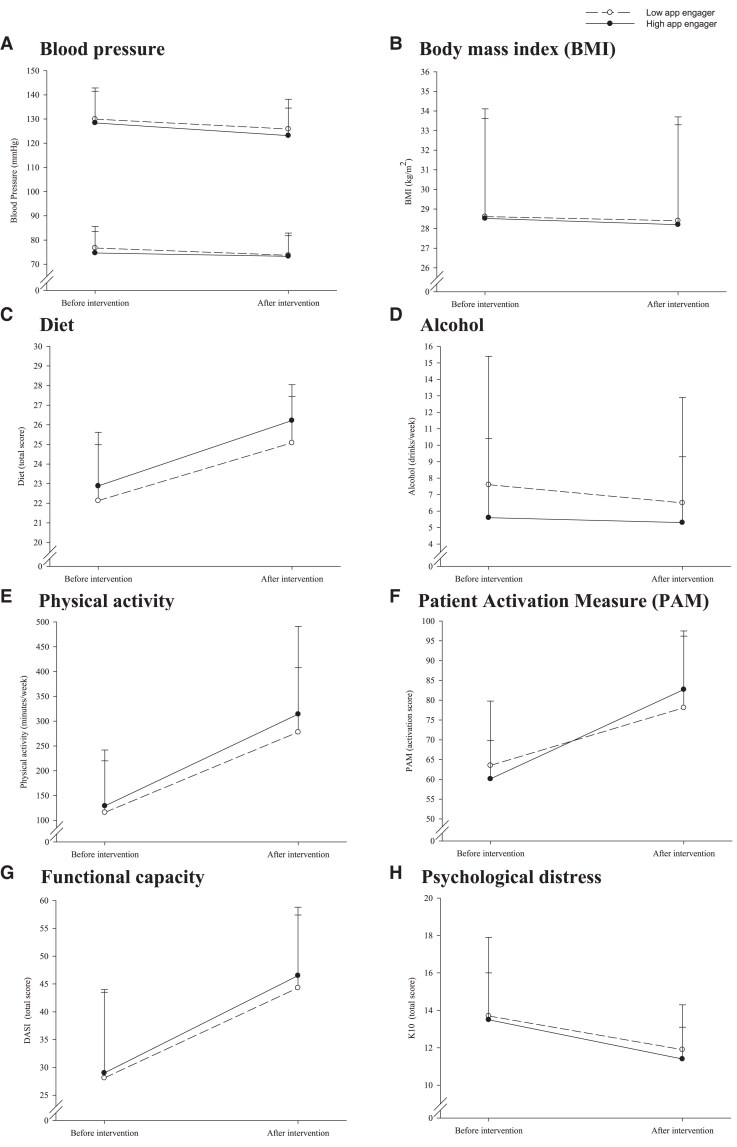
Change in risk factors from before intervention to after intervention in participants who are low app engagers (<40% engagement) and high app engagers (≥40% engagement).

Before and after intervention values according to level of app engagement are presented in *Figure 3* and summarized in Supplementary material online, *Table S3*. Following the completion of the intervention, there were significant improvements in the majority of risk factors (all *P* < 0.01) which occurred similarly in both the low and high app engager groups (*P* > 0.05). The exception was alcohol consumption (which did not change due to exposure to the intervention in any group), the PAM—whereby a greater increase over time occurred in high app engagers than in low app engagers (*P* = 0.012 for interaction), and patient’s perception of overall health when measured on a visual analogue scale—which was rated higher in low app engagers and lower in high app engagers following exposure to the intervention (*P* = 0.047).

**Figure 4:**
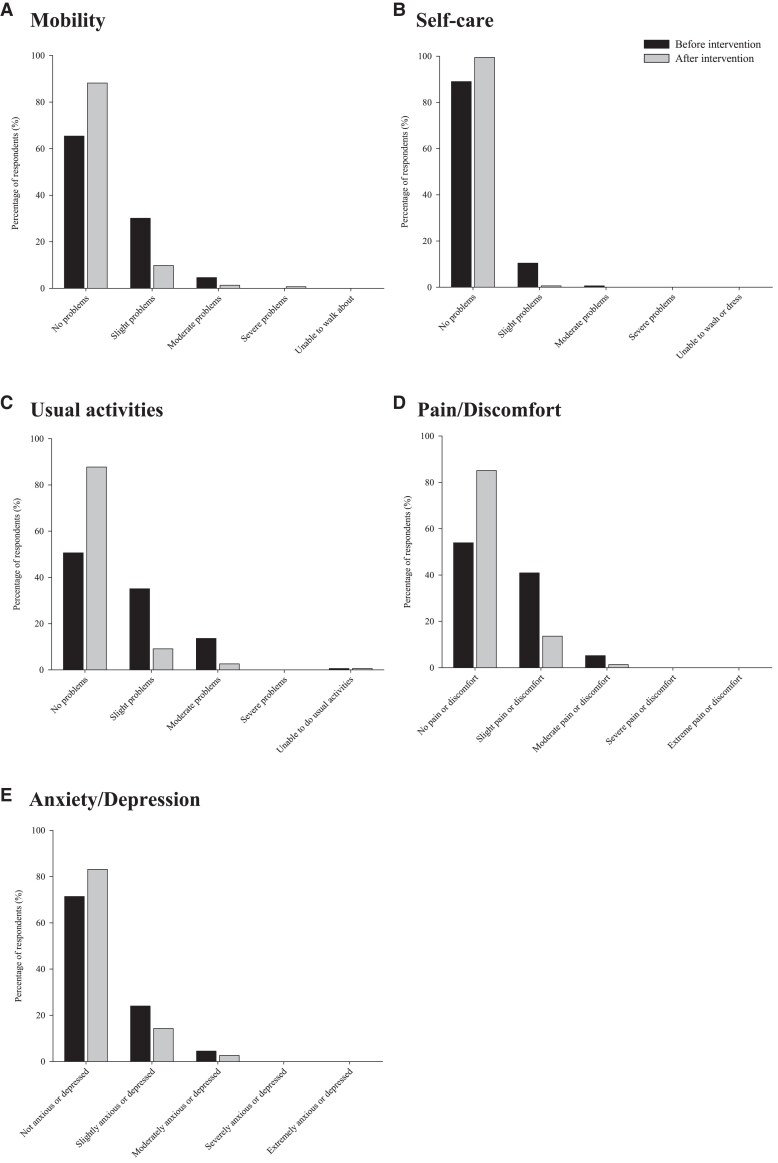
Percentage of responses by level of severity for EQ-5D-5L dimensions before and after intervention.

Health status in each of the five EQ-5D-5L dimensions before and after intervention are shown in *Figure 4*. There were significant differences in the distribution of responses, such that more patients responded that they had fewer problems after the intervention compared with the beginning for the following dimensions: mobility (panel *A*), pain/discomfort (panel *D*) and anxiety/depression (panel *E*) dimensions (all *P* < 0.001) but not self-care (panel *B*; *P* = 0.939) or usual activities (panel *C*; *P* = 0.262).

### Hospital readmissions and mortality (intervention group)

In the ‘Heart Health at Home’ cohort, 77 (56%) patients had a total of 149 readmissions for any cause within 12 months of discharge, of which 15% were readmitted within 30 days and 27% within 90 days. For cardiac readmissions, 33 (24%) patients had 56 readmissions within 12 months post-discharge, of which 8% were readmitted within 30 days and 15% within 90 days. There were no deaths in the ‘Heart Health at Home’ study cohort over the 12-month post-discharge period.

Increased physical activity from baseline to follow-up was the only significant predictor of reduced 12-month all-cause hospital readmission (see Supplementary Material 4). There were no other significant predictors of 12-month all cause readmission in the ‘Heart Health at Home’ group.

### Propensity score matching (assessing balance)

*Table 2* presents the baseline characteristics used to estimate the propensity score according to CR programme type before and after propensity score matching. It demonstrates the effectiveness of the matching process by highlighting significant differences between groups for all variables prior to matching, which were successfully balanced post-matching with no significant group differences remaining for any variable. Further, there were no statistically significant differences in baseline characteristics between matched and unmatched participants in the composition of the F2F-CR control group (see Supplementary material online, *Table S5*). The same assessment was not performed for the usual care group composition because there were very few (*n* = 5) unmatched participants to make a valid comparison.

**Table 2:** Baseline characteristics according to CR programme type before and after propensity score matching

	Pre-matching				Heart Health at Home (DeCR) matched with usual care			Heart Health at Home (DeCR) matched with F2F-CR		
	Heart Health at Home (DeCR)	F2F-CR	Usual care	*P*-value	Heart Health at Home (DeCR)	Usual care	*P*-value	Heart Health at Home (DeCR)	F2F-CR	*P*-value
Sample size	138	597	31 628	—	133	266	—	115	115	—
Age, ≥65 years (*n*, %)	89 (65)	412 (69)	25 741 (81)	<0.001	87 (65)	181 (68)	0.678	75 (65)	84 (73)	0.253
Male (*n*, %)	97 (70)	441 (74)	18 854 (60)	<0.001	93 (70)	184 (69)	0.969	84 (73)	85 (74)	1.000
IRSD, lowest 50th percentile (*n*, %)	50 (36)	120 (20)	11 368 (36)	<0.001	50 (38)	73 (27)^a^	0.051	38 (33)	24 (21)	0.053
Admission year										
2019 (*n*, %)	13 (9.4)	139 (23.3)	6532 (20.7)	0.001	12 (9.0)	22 (8.3)	0.949	10 (8.7)	10 (8.7)	1.000
2020 (*n*, %)	125 (91)	458 (77)	25 096 (79)		121 (91)	244 (92)		105 (91)	105 (91)	
Charlson comorbidity index										
Mean (SD)	1.41 (1.8)	1.64 (1.8)	2.39 (2.5)	<0.001	1.33 (1.7)	1.26 (1.6)	0.671	1.32 (1.7)	1.30 (1.6)	0.903
>1 (*n*, %)	52 (38)	232 (39)	16 563 (52)	<0.001	48 (36)	90 (34)	0.738	40 (35)	41 (36)	1.000
Re-admission risk, PEGASUS-TIMI 54										
Mean (SD)	2.41 (1.8)	2.92 (2.1)	2.39 (1.5)	<0.001	2.35 (1.8)	2.36 (1.7)	0.968	2.56 (1.8)	2.63 (1.9)	0.776
High (≥5) (*n*, %)	26 (19)	176 (30)	2227 (7)	<0.001	24 (18)	45 (17)	0.888	24 (21)	28 (24)	0.636

DeCR, digitally enabled cardiac rehabilitation; F2F-CR, face-to-face cardiac rehabilitation; IRSD, index of relative socio-economic disadvantage; SD, standard deviation.

^a^Missing cases: [usual care] (*n* = 4).

### Hospital readmissions and mortality (‘Heart Health at Home’ vs usual care)

Propensity matching identified 133 in the ‘Heart Health at Home’ group and 266 in the usual care group (1:2) to compare outcomes between these two groups. All cause and cardiac-related rehospitalization outcomes according to 30-day, 90-day, and 12-month time periods post-discharge are summarized in *Table 3*. We observed no differences between groups regarding the number of readmissions (all-cause and cardiac related) at any time point (all *P* > 0.05). For all-cause rehospitalizations, the ‘Heart Health at Home’ group had significantly fewer days spent in hospital compared with the usual care group at 30 days (71% fewer bed-days; *P* = 0.026), 90-days (71% fewer bed-days; *P* = 0.003), and within 12-months (51% fewer bed-days; *P* = 0.041) post-discharge, and at 30-days (88% fewer bed-days; *P* = 0.005) and 90-days (74% fewer bed-days; *P* = 0.017) but not within 12-months (*P* = 0.201) post-discharge for cardiac-related rehospitalizations (*Table 3*). There were no significant mortality differences between intervention patients compared with usual care patients at 30-days (*P* = 0.99) and 12-months post-discharge (*P* = 0.97) (*Table 3*).

**Table 3:** All cause and cardiac related unplanned readmissions at 30-day, 90-day, and 12-months post-discharge in the ‘Heart Health at Home’ group vs. usual care

	30-Days					90-Days					12 Months				
	‘Heart Health at Home’	Usual care	Exp. effect Size	95% CI Exp. effect size	*P*-value	‘Heart Health at Home’	Usual care	Exp. effect size	95% CI exp. effect size	*P*-value	‘Heart Health at Home’	Usual care	Exp. effect size	95% CI exp. effect size	*P*-value
All-cause															
Number (%) of patients readmitted^a^	20 (15%)	41 (15%)	—	—	1.000	36 (27%)	72 (27%)	—	—	1.000	74 (56%)	140 (53%)	—	—	0.571
Total number of readmissions^b^	22	45	0.98	0.48, 1.48	0.931	45	102	0.88	0.57, 1.19	0.484	145	284	1.02	0.82, 1.23	0.838
Hospital bed days^c^, mean (SD)	2.5 (2.3)	5.2 (6.5)	0.29	−0.03,0.61	0.026^d^	2.7 (2.7)	5.7 (7.2)	0.29	0.05,0.52	0.003^d^	4.3 (5.6)	7.2 (11.5)	0.49	0.16,0.83	0.041^d^
Total number (%) of deaths^e^	0	6 (2)	<0.001	0, <0.001	0.990	—	—	—	—	—	0	10 (4)	<0.001	0, <0.001	0.970
Cardiac-related															
Number (%) of patients readmitted^a^	10 (8%)	29 (11%)	—	—	0.347	19 (14%)	44 (17%)	—	—	0.441	32 (24%)	67 (25%)	—	—	0.827
Total number of readmissions^b^	12	30	0.80	0.26, 1.34	0.514	22	54	0.82	0.41, 1.22	0.418	54	100	1.080	0.72, 1.44	0.649
Hospital bed days^c^, mean (SD)	1.8 (1.5)	5.7 (6.7)	0.12	−0.06,0.30	0.005^d^	2.4 (3.0)	5.3 (6.2)	0.26	−0.03,0.55	0.017^d^	4.4 (6.2)	6.5 (8.4)	0.56	0.07,1.06	0.201

SD, standard deviation; Exp. Effect Size, exponential effect size.

^a^Comparison of proportions.

^b^Mixed-effect Poisson regression for readmission count.

^c^ZT negative-binomial model was used to estimate the average bed day ratio. Exponential term was used to calculate the effect size and 95% CI.

^d^Statistically significant.

^e^Mixed-effect logistic regression for mortality.

### Hospital readmissions and mortality (‘Heart Health at Home’ vs F2F-CR)

After propensity score matching, there were a total of 115 patients per group to compare outcomes of the ‘heart Health at Home’ group with the F2F-CR group (1:1). We observed no significant differences between the ‘heart health at Home’ group and F2F-CR group across all metrics assessed (all *P* > 0.05, *Table 4*).

**Table 4:** All cause and cardiac related unplanned readmissions at 30-day, 90-day, and 12-months post-discharge in the ‘Heart Health at Home’ group vs. F2F-CR

	30-Days					90-Days					12 Months				
	‘Heart Health at Home’	F2F-CR	Exp. effect size	95% CI exp. effect size	*P*-value	‘Heart Health at Home’	F2F-CR	Exp. effect size	95% CI exp. effect size	*P*-value	‘Heart Health at Home’	F2F-CR	Exp. effect size	95% CI exp. effect size	*P*-value
All-cause															
Number (%) of patients readmitted^a^	18 (16%)	13 (11%)	—	—	0.268	31 (27%)	27 (24%)	—	—	0.602	61 (53%)	63 (55%)	—	—	0.761
Total number of readmissions^b^	21	13	1.62	0.5, 2.7	0.174	37	31	1.19	0.62, 1.76	0.467	105	103	1.02	0.74, 1.3	0.890
Hospital bed days^c^, mean (SD)	3.4 (4.0)	2.1 (2.0)	2.66	−1.17,6.49	0.183	3.2 (3.7)	2.2 (2.4)	2.16	−0.61,4.94	0.239	4.1 (5.6)	3.2 (3.4)	1.32	0.36,2.28	0.457
Total number (%) of deaths^d^	0	0	0	0	1.000	—	—	—	—	—	0	1 (1)	<0.001	0, <0.001	0.990
Cardiac-related															
Number (%) of patients readmitted^a^	10 (9%)	8 (7%)	—	—	0.577	17 (15%)	17 (15%)	—	—	1.000	28 (24%)	30 (26%)	—	—	0.726
Total number of readmissions^b^	13	8	1.62	0.19, 3.06	0.280	21	18	1.167	0.43, 1.9	0.631	45	39	1.15	0.66, 1.65	0.513
Hospital bed days^c^, mean (SD)	3.3 (4.7)	2.3 (2.4)	1.90	−2.34,6.14	0.573	3.3 (4.5)	1.7 (1.8)	4.05	−3.80,11.89	0.158	4.7 (6.6)	3.1 (3.3)	1.77	−0.48,4.03	0.378

SD, standard deviation; F2F-CR, face-to-face cardiac rehabilitation.

^a^Comparison of proportions.

^b^Mixed-effect Poisson regression for readmission count.

^c^ZT negative-binomial model was used to estimate the average bed day ratio. Exponential term was used to calculate the effect size and 95% CI.

^d^Mixed-effect logistic regression for mortality.

## Discussion

This was a nationally delivered study that provides real-world evidence for integrating new technologies into CR practice.^12,13,15^ We observed improvements across most of the clinical, behavioural and lifestyle risk factors from baseline to 8-weeks follow-up, most notably increases in physical activity which was significantly associated with reduced readmission risk at 12 months. Compared with usual care, patients in the DeCR group did not experience fewer readmissions, but they did experience a reduction in the number of hospital bed days. Patients in the DeCR group had similar healthcare utilization outcomes compared with patients who undertook F2F-CR suggesting that the intervention may be as useful as F2F-CR programmes. We observed a modest level of engagement with the SmartCR app; patients achieved an average of 40% of all their required mobile app assignments, with a higher level of engagement for tasks set (47%) compared with medication reminders (37%). We found that higher app engagers had greater improvements in activation levels, signalling increased knowledge, skills and confidence in self-managing their health. Albeit, the amount of app engagement did not affect other risk factors or readmission outcomes.

Uptake of our intervention was satisfactory and pleasing (59% of patients who met the key study requirements gave consent to participate) and adherence to complete the final follow-up assessment was high (91%), indicating that it was well accepted by patients. Historically, CR programme dropout rates are as high as between 20% and 40%.^7,29^ We speculate that adherence to complete the programme was enhanced via regular patient-nurse communication, remote monitoring, automated physical activity prescription and personalized education.^15^ Our participation rates were also substantially better than those currently reported globally for attending CR programmes^7,30,31^ or other DeCR programmes.^32^ However, we excluded a proportion (16%) of patients due to limited digital technology or low digital competence. These are two commonly reported barriers to DeCR programmes^33^ indicating that greater access and technological support to use digital health tools is needed to enable equal opportunity to participate in these types of programmes, for all patients. Also, as shown from our data, some patients preferred F2F-CR.^33^ CR availability is scarce and underutilized.^6^ Providing more CR delivery options will increase access to and participation for populations who otherwise may not attend, such as those from lower socioeconomic status groups and people living in non-urban areas.^6^ Many of these populations have higher rates of CVD and less access to cardiac services,^8^ thus compounding health disparities. However, many patients also lack access to the required technology or reliable internet connection to participate in DeCR programmes.^34^ Therefore, current DeCR programmes will not solve the service gap for all populations and further research is required to address technological inequity by reducing the digital divide that exacerbates health disparities.^34^

The DeCR intervention in our study improved patient’s CVD risk factors and these findings replicate those shown by many other digital health secondary prevention interventions.^35^ In particular, physical activity levels more than doubled from baseline to follow-up and this was associated with reduced risk of readmission, potentially via the impact on inflammation or insulin sensitivity.^36^ All individuals participating in a CR programme are encouraged to increase their exercise and physical activity levels.^37^ A limiting factor of our DeCR programme, conducted by telephone only, was the inability of an in-person assessment or video monitoring for implementation of an individualized exercise programme and thereafter, unsupervised exercise and physical activity. However, this study provides evidence that a somewhat conservative walking plan by SmartCR as part of the DeCR intervention was associated with an increase in physical activity, which could potentially result in health benefits over the longer-term, if maintained.

Overall, our level of app engagement (40%) surpasses industry benchmarks of an average rate of response of 23% and 90-day retention rate of 39%.^38^ This achievement may be explained by the SmartCR app characteristics (e.g. appropriate reminders combined with educational support), as well as input from nurses in the app-based intervention, all of which promote app engagement.^39,40^ Nevertheless, this level of app engagement was modest, particularly for medication reminders (37%). A high average frequency of seven daily medication reminders and four daily task alerts may have caused notification irritation and contributed to disengagement with the app over time, especially for more frequently recurring medication reminders. Comparing this finding with previous published literature is difficult however due to the lack of common definitions in measuring app engagement, particularly as a function of each individual app component, and heterogeneity in patient population groups and app characteristics/functionality.^24,39^

Despite these difficulties, user engagement in our study was assessed objectively via app analytics and categorized by the frequency that patients were expected to engage (e.g. percentage of assigned tasks that were achieved), rather than general usage (e.g. number of log-ins). Nonetheless, there was little evidence in our study to indicate that higher app usage equates to greater improvements in outcomes, and it was not an independent predictor of the doubling of physical activity levels seen upon completion. Limited evidence for an association between increased app user engagement and CVD risk factors and outcomes (beyond improvements in body weight) is consistent with findings from a systematic review by Spaulding *et al.*^24^ Absence of a dose response relationship between app engagement and outcomes may be attributed to many factors. Firstly, it may be possible that *some* amount of app exposure/engagement, greater than was shown by participants in our study, is required to achieve significant changes in CVD health behaviours, risk factors and outcomes. Secondly, we did not conduct power analyses to detect significant relationships, as the cohort size was determined by participant enrolments during the study period. Thirdly, our study design did not enable us to investigate the reasons for the amount of app usage by participants, which may arise from issues with the SmartCR app itself or from the types of individuals who use it. Likewise, we demonstrated an interaction effect, such that patient’s perception of their overall health changed depending on the influence of level of app engagement (low vs. high), yet this relationship cannot infer causation and responses may be influenced by other personal or health-related factors. Additionally, high app engagers may have had poorer health and in turn engaged more with the app. Delineating between these justifications requires independent research of mobile app testing and the diverse perspectives of the target audience.

The app is one component of the intervention, and findings cannot be attributed to the technology alone. A series of nurse telehealth consultations were provided routinely throughout the duration of the intervention which cannot be dissociated from the SmartCR app. We speculate that the beneficial effect of our intervention is due to the ‘sum of its parts’ including personalized nurse consultations integrated with remote monitoring and patient self-management via the app. Combining digital technologies, particularly remote monitoring, with methods to enhance patient-clinician interaction, appear to be the core drivers of effective outcomes in other DeCR interventions.^15,16^

We extended upon the previous study by Varnfield^14^ (using the same SmartCR app), in addition to other research assessing DeCR interventions,^41^ by evaluating the likely benefit on hospital utilization and mortality of a DeCR programme. Despite including a mix of index cardiac admissions, there was no bias for observed rehospitalization and the index admission was not predictive of readmissions. Our results suggest that DeCR is associated with improved health care utilization compared with usual care; however, a properly powered experiment is required to detect this effect. Patients in our intervention had similar rehospitalization rates compared with usual care but the intervention was associated with significantly fewer days spent in hospital. This contributes to the growing body of evidence demonstrating that DeCR programmes reduce hospital utilization compared with usual care.^42^ However, our findings align with systematic review evidence that consistently shows no mortality benefits from DeCR programmes.^42^ Although the rehospitalization rates were similar between the groups, we speculate that the intervention in our study may have reduced the severity of rehospitalization, although more detailed information about the cause for rehospitalization would be required to confirm or deny this assertion. We hypothesize that by virtue of more easy access to nurses and/or nurse-guided education and support, patients attended hospital earlier and at a lower acuity than if they were to wait, resulting in readmission similarities between the groups, yet shorter length of stay in the intervention group.

We also observed that the DeCR intervention in our study performed similarly compared with F2F-CR across all metrics and this is consistent with prior studies.^35,43^ We found no group differences in rehospitalization, days spent in hospital and mortality outcomes between our intervention and F2F-CR. This corroborates the existing evidence that DeCR programmes provide similar benefits to F2F-CR with respect to outcomes, whilst providing additional benefits of increased access, adherence and patient choice for those with digital technology capabilities and competence.^35^

### Strengths and limitations

Our study had excellent uptake and low dropout rates, thus providing greater assurance for the acceptance of DeCR programmes in practice. Recruitment was national, albeit a representative sample was not recruited in each State and Territory. Due to our recruitment processes and desire for high response rates, particularly during the COVID-19 pandemic, we found that females were under-represented compared with males. Whilst this observation resembles broader gender disparities in the diagnosis, treatment and CR attendance in females, it underscores the importance for targeted research that includes more females. The index cardiac hospitalizations were varied, yet all were eligible for CR and the intervention, including the SmartCR app, also aligned with the core components of a phase 2 CR programme.^17^ The burden of illness in the study population appeared to be mild, based on the Charlson comorbidity index and length of stay, indicating that involvement and results from our intervention may not extend to more medically complex individuals or people with heart failure.

Given the opportunity to afford private health care, it may be assumed that the cohort were of mid-to-high socio-economic status, however more than one third of patients in the DeCR group resided in regional or rural locations and lower socioeconomic areas (bottom 50th percentile). We excluded participants who lacked access to a smart phone and internet connection which may have introduced selection preferences by including individuals who were agreeable to engage with a digital health intervention. Additionally, the level of support provided to participants in the intervention arm may have contributed to a Hawthorne effect, whereby both participants and nurses altered their behaviours due to increased monitoring and structured interaction. Together, these biases could have favourably affected uptake and completion rates of the study. Furthermore, whilst the COVID-19 pandemic highlighted the need for remotely delivered CR programmes, lockdown restrictions in some parts of Australia during the study period may also have contributed to the admirable uptake rate to our intervention.

Propensity score methods have previously been used to assess similar real-world CR studies^44,45^ but have several disadvantages. It cannot control for any unknown confounders or baseline characteristics which were either not measured or not included in the propensity score model (such as frailty, polypharmacy, prior admissions and socioeconomic status). However, we selected this study design for the following advantages. Firstly, a prior feasibility and effectiveness randomized controlled trial investigating a similar model of care had already been conducted.^14^ We wanted to extend upon this prior research by implementing the programme nationally in an uncontrolled setting, and to assess its association with longer-term outcomes including hospital utilization and mortality. Secondly, there is a growing call for more DeCR real-world studies.^12,13,15^ This study design provided us with a method for investigating the complexity of real-world conditions and a more nuanced understanding of potential benefits as they occur in everyday life, with minimal data collection burden. Due to data privacy and security restrictions, access to hospital readmission data by investigators was contained to matched participant pairs only and the probability of outcomes in the non-propensity matched individuals could not be evaluated. Owing to the pre–post study design, some of the improvements in risk factors may have occurred as a natural consequence of the recovery process over time. There also was potential for self-report bias when asking participants to complete outcome measures, which may be exemplified in entirely decentralized studies without any physical contact between researchers and participants. Integrating telemonitoring devices could help avoid or mitigate these issues. A hybrid trial would also enable some study activities that are important for study endpoints or outcomes to occur on-site or by seeking the support of pharmacy, GPs or other allied health professionals.

The application of our intervention was in a private health care setting whereupon approximately 55% of Australia’s 26 million population have private health insurance.^46^ CR is an outpatient service that is not covered by a universal (Medicare) health scheme but is subsidized by private health insurance for individuals with the appropriate coverage level. Considering the $14.3 billion per annum expenditure attributed to CVD that is borne by government and private health insurers,^47^ it was pertinent to test the benefits of a new intervention to help determine whether to continue allocating limited resources to the ‘Heart Health at Home’ DeCR programme for eligible members. The overall findings enhance the existing level of available evidence about DeCR programmes and suggest that the intervention could be applied beyond what worked in a private health care context to different populations with similar usefulness, to benefit more individuals. This is the focus of the Risk-Guided CAD trial to test a nurse-led, technology-enabled model of health care delivery in coronary artery disease patient compared with standard care (ClinicalTrials.gov NCT04966117).

### Implications for clinical practice and future research

Participating in CR, regardless of the method, improves risk factors and health outcomes and lowers healthcare utilization. The ‘Heart Health at Home’ programme generates evidence for health care providers to adopt DeCR to support patient care, wherever practical and/or possible. However, despite the growing evidence from a predominantly clinical perspective, adoption of DeCR is slow and is not a remedy to address the large unmet demand for participation in CR. There are constraints to be attended to before large scale implementation into clinical practice within the healthcare ecosystems in which they operate, including but not limited to funding for the technology and health workforce resourcing. Understanding the factors influencing implementation of DeCR from a health care system perspective is required. In addition, future well-designed and appropriately powered randomized controlled trials that are not targeted towards privately insured individuals (such as the ongoing Risk-Guided CAD trial) are required to evaluate DeCR programme impacts and determine non-inferiority to traditional styles of CR. Cost effectiveness analysis is required to establish cost savings from a patient, health system and societal level. These recommendations are in addition to our other suggestions for future research to address inequalities in access to digital health (such as socio-economic disadvantage and limited digital literacy), ensure greater representation of females in digital health research to close the gender gap in heart health, and better evaluate app user experiences. This research is necessary to inform the development of targeted strategies, funding and practice recommendations to scale up to increase CR uptake, particularly in under-represented groups.

## Conclusion

In conclusion, for those undertaking it, our DeCR intervention improved clinical risk factors and healthy lifestyle changes. It is possible that DeCR has the ability to reduce healthcare utilization, akin to F2F-CR, but more formal assessment is required. The results give some assurance for broader application of digitally integrated programmes in an online world and beyond the private health care sector as an opportunity to break down barriers and connect with patients without the constraint of physical geography. Offering DeCR as a post-discharge option for cardiac patients warrants further investigation.

## Supplementary Material

ztaf043_Supplementary_Data

## Data Availability

The data underlying this article will be shared on reasonable request to the corresponding author.
