# Supplementary material for: Effects of a digitally enabled cardiac rehabilitation intervention on risk factors, recurrent hospitalization and mortality
Source: Eur Heart J Digit Health. 2025 Apr 29;6(4):688–703. doi: 10.1093/ehjdh/ztaf043 (PMC12282376; doi:10.1093/ehjdh/ztaf043)
Supplement: ztaf043_Supplementary_Data [file ztaf043_supplementary_data.docx]

Supplementary file

## **Supplementary material 1**

Cardiovascular diagnosis and/or procedures eligible for cardiac rehabilitation, as defined by the National Heart Foundation of Australia.

- Acute Myocardial Infarction (MI) – both ST elevation and Non-ST elevation; including patients with and without post-MI revascularisation, e.g. those medically-managed only
- Revascularisation procedures o Coronary artery bypass graft surgery o Percutaneous coronary interventions
- Medically-managed coronary artery disease, e.g. stable angina
- Valve device, replacement and repair
- Permanent pacemaker and implantable defibrillator insertion
- Heart transplant
- Atrial fibrillation
- Those at high risk for coronary artery disease
- Other vascular or heart diseases and interventions
- Familial hypercholesterolaemia

*Source: Defined by the National Heart Foundation of Australia and listed in the Australian Cardiovascular Health and Rehabilitation Association (ACRA)* [*Core Components*](https://www.heartlungcirc.org/article/S1443-9506(14)00822-1/pdf) *of Cardiovascular Disease Secondary Prevention and Cardiac Rehabilitation (2014).*

## **Supplementary Table 2**

Codes used to define eligible cardiovascular diagnoses and procedures.

| **Code List** | | |
| --- | --- | --- |
| **Ranking** | **Code** | **Definition** |
| **1** | **AMI - STEMI** |  |
|  | I21.0 | Acute transmural myocardial infarction of anterior wall |
|  | I21.1 | Acute transmural myocardial infarction of inferior wall |
|  | I21.2 | Acute transmural myocardial infarction of other sites |
|  | I21.3 | Acute transmural myocardial infarction of unspecified site |
|  |  |  |
| **2** | **AMI - Non-STEMI** |  |
|  | I21.4 | Acute subendocardial myocardial infarction |
|  | I21.9 | Acute myocardial infarction, unspecified |
|  |  |  |
| **3** | **Unstable Angina** |  |
|  | I20.0 | Unstable angina |
|  |  |  |
| **4** | **Coronary Artery Bypass Graft** |  |
|  | 38497-00 | Coronary artery bypass, using 1 saphenous vein graft |
|  | 38497-01 | Coronary artery bypass, using 2 saphenous vein grafts |
|  | 38497-02 | Coronary artery bypass, using 3 saphenous vein grafts |
|  | 38497-03 | Coronary artery bypass, using ≥ 4 saphenous vein grafts |
|  | 38497-04 | Coronary artery bypass, using 1 other venous graft |
|  | 38497-05 | Coronary artery bypass, using 2 other venous grafts |
|  | 38497-06 | Coronary artery bypass, using 3 other venous grafts |
|  | 38497-07 | Coronary artery bypass, using ≥ 4 other venous grafts |
|  | 38500-00 | Coronary artery bypass, using 1 LIMA graft |
|  | 38503-00 | Coronary artery bypass, using ≥ 2 LIMA grafts |
|  | 38500-01 | Coronary artery bypass, using 1 RIMA graft |
|  | 38503-01 | Coronary artery bypass, using ≥ 2 RIMA grafts |
|  | 38500-02 | Coronary artery bypass, using 1 radial artery graft |
|  | 38503-02 | Coronary artery bypass, using ≥ 2 radial artery grafts |
|  | 38500-03 | Coronary artery bypass, using 1 epigastric artery graft |
|  | 38503-03 | Coronary artery bypass, using ≥ 2 epigastric artery grafts |
|  | 38500-04 | Coronary artery bypass, using 1 other arterial graft |
|  | 38503-04 | Coronary artery bypass, using ≥ 2 other arterial grafts |
|  | 38500-05 | Coronary artery bypass, using 1 composite graft |
|  | 38503-05 | Coronary artery bypass, using ≥ 2 composite grafts |
|  | 90201-00 | Coronary artery bypass, using 1 other graft, not elsewhere classified |

|  | 90201-01 | Coronary artery bypass, using 2 other grafts, not elsewhere classified |
| --- | --- | --- |
|  | 90201-02 | Coronary artery bypass, using 3 other grafts, not elsewhere classified |
|  | 90201-03 | Coronary artery bypass, using ≥ 4 other grafts, not elsewhere classified |
|  |  |  |
| **5** | **Angioplasty** |  |
|  | 38300-00 | Percutaneous transluminal balloon angioplasty of 1 coronary artery |
|  | 38303-00 | Percutaneous transluminal balloon angioplasty of ≥ 2 coronary arteries |
|  | 38300-01 | Open transluminal balloon angioplasty of 1 coronary artery |
|  | 38303-01 | Open transluminal balloon angioplasty of ≥ 2 coronary arteries |
|  | 38306-00 | Percutaneous insertion of 1 transluminal stent into single coronary artery |
|  | 38306-01 | Percutaneous insertion of ≥ 2 transluminal stents into single coronary artery |
|  | 38306-02 | Percutaneous insertion of ≥ 2 transluminal stents into multiple coronary arteries |
|  | 38306-03 | Open insertion of 1 transluminal stent into single coronary artery |
|  | 38306-04 | Open insertion of ≥ 2 transluminal stents into single coronary artery |
|  | 38306-05 | Open insertion of ≥ 2 transluminal stents into multiple coronary arteries |
|  |  |  |
| **6** | **Coronary Artery Disease** |  |
|  | I25.10 | Atherosclerotic heart disease of unspecified vessel |
|  | I25.11 | Atherosclerotic heart disease of native coronary artery |
|  | I25.12 | Atherosclerotic heart disease of autologous bypass graft |
|  | I25.13 | Atherosclerotic heart disease of nonautologous bypass graft |
|  |  |  |
| **7** | **Angina** |  |
|  | I20.1 | Angina pectoris with documented spasm |
|  | I20.8 | Other forms of angina pectoris |
|  | I20.9 | Angina pectoris, unspecified |
|  |  |  |
| **8** | **Hypercholesterolaemia** |  |
|  | E78.0 | Pure hypercholesterolemia, unspecified |
|  |  |  |
| **9** | **Valve** |  |
|  | 38483-00 | Decalcification of aortic valve leaflet |
|  | 38270-01 | Percutaneous balloon aortic valvuloplasty |
|  | 38475-02 | Aortic valve annuloplasty |
|  | 38477-02 | Aortic valve annuloplasty with ring insertion |
|  | 38480-00 | Repair of aortic valve, 1 leaflet |
|  | 38481-00 | Repair of aortic valve, ≥ 2 leaflets |
|  | 38488-08 | Percutaneous replacement of aortic valve with bioprosthesis |
|  | 38488-00 | Replacement of aortic valve with mechanical prosthesis |
|  | 38488-01 | Replacement of aortic valve with bioprosthesis |
|  | 38489-00 | Replacement of aortic valve with homograft |
|  | 38489-01 | Replacement of aortic valve with unstented heterograft |
|  |  |  |
| **10** | **Permanent Pacemaker** |  |
|  | 38353-00 | Insertion of cardiac pacemaker generator |
|  | 38393-00 | Insertion of cardiac defibrillator generator |
|  |  |  |
| **11** | **Atrial Fibrillation** |  |
|  | I48.0 | Paroxysmal atrial fibrillation |
|  | I48.1 | Persistent atrial fibrillation |
|  | I48.2 | Chronic atrial fibrillation |
|  | I48.3 | Typical atrial flutter |
|  | I48.4 | Atypical atrial flutter |
|  | I48.9 | Atrial fibrillation and atrial flutter, unspecified |
|  |  |  |
| **12** | **Heart Failure and Cardiac Devices / Cardiomyopathy** |  |
|  | I50.0 | Congestive heart failure |
|  | I50.1 | Left ventricular failure |
|  | I50.9 | Heart failure, unspecified |
|  | I42.0 | Dilated cardiomyopathy |
|  | I42.1 | Obstructive hypertrophic cardiomyopathy |
|  | I42.2 | Other hypertrophic cardiomyopathy |
|  | I42.3 | Endomyocardial (eosinophilic) disease |
|  | I42.4 | Endocardial fibroelastosis |
|  | I42.5 | Other restrictive cardiomyopathy |
|  | I42.6 | Alcoholic cardiomyopathy |
|  | I42.7 | Cardiomyopathy due to drugs and other external agents |
|  | I42.8 | Other cardiomyopathies |
|  | I42.9 | Cardiomyopathy, unspecified |
|  | Z95.0 | Presence of Cardiac Pacemaker |
|  |  |  |
| **13** | **Heart Transplant** |  |
|  | 90205-00 | Heart transplantation |

## **Supplementary Table 3**

Change in risk factors and health and lifestyle behaviours before and after completion of the Heart Health at Home DeCR program.

|  | **N** | **Before intervention** | **me** | **After intervention** **an (SD)** | **p-value for interaction** |
| --- | --- | --- | --- | --- | --- |
| ***Risk factors*** |  |  |  |  |  |
| Systolic blood pressure, mmHg  Low app engagers (<40%)  High app engagers (≥40 %) | 67  67 | 130 (12)  128 (14) |  | 126 (12)  123 (11) | 0.590 |
| Diastolic blood pressure, mmHg  Low app engagers (<40%)  High app engagers (≥40 %) | 67  67 | 77 (9)  75 (9) |  | 74 (9)  73 (9) | 0.264 |
| Body mass index, kg/m^2^  Low app engagers (<40%)  High app engagers (≥40 %) | 73  68 | 28.6 (5.5)  28.5 (5.1) |  | 28.4 (5.3)  28.2 (5.1) | 0.434 |
| Diet score  Low app engagers (<40%)  High app engagers (≥40 %) | 77  73 | 22.1 (2.9)  22.9 (2.7) |  | 25.1 (2.4)  26.2 (1.8) | 0.380 |
| Alcohol, drinks per week  Low app engagers (<40%)  High app engagers (≥40 %) | 31  39 | 7.6 (7.8)  5.6 (4.8) |  | 6.5 (6.4)  5.3 (4.0) | 0.398 |
| Physical activity, minutes per week  Low app engagers (<40%)  High app engagers (≥40 %) | 69  68 | 116 (104)  129 (113) |  | 278 (130)  313 (177) | 0.319 |
| Medication adherence, MMAS  Low app engagers (<40%)  High app engagers (≥40 %) | 77  72 | 0.22 (0.58)  0.25 (0.52) |  | 0.01 (0.11)  0 00 (0.00) | 0.647 |
|  |  |  |  |  |  |
| ***Health and lifestyle behaviours*** | |  |  |  |  |
| Functional capacity, DASI  Low app engagers (<40%)  High app engagers (≥40 %) | 79  76 | 28.1 (15.9)  29.0 (14.5) |  | 44.3 (14.5)  46.5 (10.9) | 0.587 |
| Psychological distress, K10  Low app engagers (<40%)  High app engagers (≥40 %) | 78  75 | 13.7 (4.2)  13.5 (2.5) |  | 11.9 (2.4)  11.4 (1.7) | 0.560 |
| Patient engagement, PAM  Low app engagers (<40%)  High app engagers (≥40 %) | 79  74 | 63.5 (16.3)  60.1 (9.7) |  | 78.1 (18.1)  82.7 (14.8) | 0.012* |
|  |  |  |  |  |  |

|  | **N** | **Before intervention** | **After intervention** | **p-value for interaction** |
| --- | --- | --- | --- | --- |
| ***Health related quality of life*** | |  |  |  |
| EQ-5D-5L (Visual analogue scale)  Low app engagers (<40%)  High app engagers (≥40 %) | 78  75 | 70 (20)  70 (15) | 76 (20)  67 (19) | 0.047* |
| EQ-5D-5L (Index value - AUS)  Low app engagers (<40%)  High app engagers (≥40%) | 77  75 | 0.939 (0.071)  0.943 (0.063) | 0.977 (0.051)  0.982 (0.034) | 0.877 |

mmHg: millimetres of mercury;

## **Supplementary Material 4**

Results from the binary logistic regression, variables that predict 12-month readmission.

|  |  | **Model Summary** |  |
| --- | --- | --- | --- |
|  | -2 Log likelihood | Cox & Snell R Square | Nagelkerke R Square |
| Step 1 | 65.241 | .098 | .131 |
| Step 2 | 60.446 | .179 | .239 |

|  |  | **Odds Ratio** | **95% Confidence Interval for Odds Ratio** | |
| --- | --- | --- | --- | --- |
|  |  |  | Lower | Upper |
| Step 1 | Physical activity *minutes per week before*  *intervention* | .994 | .988 | 1.000 |
| Step 2 | Physical activity *minutes per week before*  *intervention* | .993 | .988 | .999 |
|  | Change in physical activity from before to after the intervention (minutes per week) | .994 | .989 | 1.000 |
